# Secular Slowing of Auditory Simple Reaction Time in Sweden (1959–1985)

**DOI:** 10.3389/fnhum.2016.00407

**Published:** 2016-08-18

**Authors:** Guy Madison, Michael A. Woodley of Menie, Justus Sänger

**Affiliations:** ^1^Department of Psychology, Umeå UniversityUmeå, Sweden; ^2^Department of Psychology, Technische Universität ChemnitzChemnitz, Germany; ^3^Center Leo Apostel for Interdisciplinary Studies, Vrije Universiteit BrusselBrussels, Belgium

**Keywords:** reaction time, intelligence, secular trend, auditory reaction time, evolution, human evolution

## Abstract

There are indications that simple reaction time might have slowed in Western populations, based on both cohort- and multi-study comparisons. A possible limitation of the latter method in particular is measurement error stemming from methods variance, which results from the fact that instruments and experimental conditions change over time and between studies. We therefore set out to measure the simple auditory reaction time (SRT) of 7,081 individuals (2,997 males and 4,084 females) born in Sweden 1959–1985 (subjects were aged between 27 and 54 years at time of measurement). Depending on age cut-offs and adjustment for aging related slowing of SRT, the data indicate that SRT has increased by between 3 and 16 ms in the 27 birth years covered in the present sample. This slowing is unlikely to be explained by attrition, which was evaluated by comparing the general intelligence × birth-year interactions and standard deviations for both male participants and dropouts, utilizing military conscript cognitive ability data. The present result is consistent with previous studies employing alternative methods, and may indicate the operation of several synergistic factors, such as recent micro-evolutionary trends favoring lower *g* in Sweden and the effects of industrially produced neurotoxic substances on peripheral nerve conduction velocity.

## Introduction

It has recently been suggested that physiological measures of cognitive ability, such as simple reaction time (SRT), may be immune to the Flynn effect – the increase in psychometric intelligence (IQ) of three points per decade (Pietschnig and Voracek, 2015). This results from the fact that such measures serve as *biomarkers* of the underlying general intelligence factor *(g)* and are therefore capable of consistently measuring the same cognitive parameter across cohorts, i.e., processing speed (Jensen, 2011). In contrast, pencil-and-paper tests of IQ fail to precisely and invariantly measure the same dimension of performance across cohorts. It is this lack of test invariance that is thought to in part give rise to the Flynn effect, which is characterized by heterogeneous gains among test specificities rather than in substantive latent factors, such as *g* (Wicherts et al., 2004). The secular trend characteristics of SRT and other elementary cognitive tasks (ECTs) are therefore of considerable theoretical interest, as they may in some cases reveal important information about the secular trend characteristics of *g* (Gottfredson, 2007).

Various lines of evidence support the argument that secular trends on simple ECT performance may in fact be quite discrepant from the Flynn effect on conventional psychometric tests. For example, Nettelbeck and Wilson (2004) found no indication of secular gains on *inspection time* performance over 30 years in an Australian sample, despite indications of concurrent gains on a test of verbal ability administered to the same cohorts. Simple timing tasks (i.e., finger tapping) similarly reveal no Flynn effect, despite concurrent gains on the *Trail Making* and the *Symbol Digit* tests (Dickinson and Hiscock, 2011). A meta-analytic study by Silverman (2010) revealed apparent long term declines in performance on visual *simple reaction time* (SRT) across a handful of Western countries between the 1880s and the 2000s. Woodley et al. (2013) reanalyzed Silverman’s data utilizing meta-regression, finding evidence of a robust secular trend toward slowing SRT between the years 1889 and 2004, amounting to a lag increase of 77 ms. This finding was met with such skepticism that six commentaries advancing a range of criticisms were published in response (Dodonova and Dodonov, 2013; Flynn, 2013; Silverman, 2013; Nettelbeck, 2014; Parker, 2014; Woods et al., 2015). A common theme among these commentaries concerned the possibility of confounding effects stemming from the presence of uncontrolled *methods variance*, i.e., variation in the results of studies due to variations in the instruments and in the experimental protocols used to measure SRT (Jensen, 2006, 2011). Dodonova and Dodonov (2013) even estimated an alternative and shallower secular trend in SRT based on a set of corrections for method-related increases in the measured SRTs. A different application of these corrections, coupled with a tightening of the inclusion rules so as to exclude non-Anglosphere data points from consideration, established the presence of secular slowing trends amounting to between -34.49 ms and -44.6 ms across 115 years, but with much improved fit, as indicated by lower heterogeneity among the studies (Woodley et al., 2014b). Much controversy surrounded the use of Galton’s nineteenth century population SRT values, which were confounded by a small calibration error in the chronoscope and also possibly by his selecting only the fastest of three trials per participant. On this basis Dodonova and Dodonov (2013) proposed that Galton’s sample might have had a mean SRT of around 207 ms, rather than 188 ms. Thresholding analysis revealed however that Galton’s sample could have been slower still (213 ms), and the secular decline trend detected in Woodley et al. (2014b) would still hold (Woodley of Menie et al., 2015a). A more recent reanalysis of the Silverman data in which only four large and representative UK studies (*N*s > 500) were corrected for methods variance, and in which Galton’s sample was assigned a mean SRT of 208.5 ms, revealed a secular slowing amounting to -22.8 ms across a century (Woodley of Menie et al., 2015b). A similar methods variance corrected cross-study comparison involving four normalization samples indicates secular performance declines in another ECT – color acuity (Woodley of Menie and Fernandes, 2016).

Despite the apparent robustness of the results of Silverman’s original study, Dodonova and Dodonov’s (2013) criticisms nonetheless highlighted the importance of a lack of standardization in methods as a genuine source of uncertainty in estimating secular changes in SRT using cross-study comparisons. One solution to this problem would be to record SRTs with common instrumentation and methods from age-matched cohorts over the course of at least a few decades, obviously a major undertaking. While such data exist for inspection times (Nettelbeck and Wilson, 2004) and finger tapping (Dickinson and Hiscock, 2011), to the best of our knowledge no such data yet exist for SRT.

Another solution is to obtain both cross-sectional and longitudinal SRT data from participants covering a wide age range. The ratio of the two trends can be used to infer the presence of secular trends on the basis that steeper longitudinal relative to cross-sectional slowing trends would indicate that older cohorts were relative faster than younger ones when matched for age, suggesting a secular decline in performance, and vice-versa in the case of secular gains (Verhaeghen, 2014). Based on an analysis of nine studies reporting both cross-sectional and longitudinal change data for a variety of processing speed measures, Verhaeghen (2014) concludes that the complexity of the measure seems to increase the sensitivity of the measure to secular gains. A more complex processing speed measure such as *perceptual speed* is, for example, associated with ratios >1, indicating that the longitudinal trend is shallower than the cross-sectional trend, and that younger cohorts are relatively faster than older ones when matched for age based on trend extrapolation. By contrast simple measures of processing speed (such as *Go/No Go*) are associated with ratios <1, indicating potential secular declines. In a reanalysis of Verhaeghen’s (2014) data on SRT, it was found that the weighted average ratio across three studies was 0.90 (Woodley of Menie et al., 2015b). Verhaeghen (2014, p. 256) himself reports combined ratios of 0.85 for SRT and 0.97 for choice RT. In any case, these findings are consistent with modest SRT, and possibly also CRT latency increases across generations. A reanalysis of the study of Deary and Der (2005), utilizing a curve-fitting variant of Verhaeghen’s method, found evidence of significant secular declines among the female, but not the male cohorts (Woodley et al., 2014a). As more complex processing speed measures are multi-factorial (they tap decision time and certain memory and attention components in addition to pure processing speed; Jensen, 2006), improvements with respect to these other, potentially more trainable performance determinants may mask secular declines at the level of processing speed on such measures, which might account for Verhaeghen’s (2014) observation of a positive association between ECT measure complexity and Flynn effect sensitivity.

Large-scale cross-sectional studies investigating measures of SRT have found that age related slowing is not homogeneous across the range of ages, with performance typically decreasing more rapidly among older cohorts. For example, Jaworski et al. (2011) found no significant aging effects on auditory SRT when the mean of a group of 21- to 35-year-olds were compared with a group of 36- to 55-year-olds, with the differences becoming much more pronounced and significant when both of the groups were compared with a group of 56- to 80-year-olds. Similar observations have been made by Der and Deary (2006, p. 65) using cross-sectional comparison of performance on visual SRT, with performance among those younger than 50 showing little variation as a function of age, and sharp performance declines occurring among those older than 50. One explanation for this heterogeneity, consistent with Verhaeghen’s (2014) results, is that period effects (i.e., in this case secular slowing in the speed of SRT) are canceling out the longitudinal age-related slowing among the younger cohorts. In other words people in their 40s are now approximately *as slow* as people in their 20s *after* experiencing age-related slowing, indicating that they were relatively faster when younger. Older cohorts (i.e., those >50 years of age) may experience much more pronounced age-related slowing (e.g., Deary and Der, 2005), which offsets the period effect, yielding an apparent cross-sectional decline in performance among this group. Directly adjusting cross-sectional data for the longitudinal trend might therefore bring out the secular slowing trend.

To investigate this possibility, data on auditory SRT from 7,081 Swedish individuals born between 1959 and 1985 were collected in connection with a large web-based data collection that included a wide range of instruments and questionnaires. The cross-sectional data were adjusted for longitudinal age-related auditory SRT slowing using estimates derived from Fozard et al. (1994), in order to establish the presence of a secular trend. Remarkably, however, even the non-adjusted SRT data revealed clear indications of *increasing* SRT performance with age among the cohorts aged 27–50, which is the range that appears to be ‘static’ in terms of slowing in cross-sectional studies of SRT performance (e.g., Der and Deary, 2006; Jaworski et al., 2011). On this basis it was decided to present both aging-adjusted and non-adjusted SRT data.

## Materials and Methods

### Participants

Data were collected online from October 2012 to May 2013 from the STAGE cohort of twins born between 1959 and 1985 (Lichtenstein et al., 2006). This cohort is part of the Swedish Twin Registry (STR), and has been involved in one previous data collection wave that took place in 2005–2006. The STR includes all twins born in Sweden since 1886 (Lichtenstein et al., 2002 and Lichtenstein et al., 2006 for further details on the STAGE cohort and its representativity). The study was approved by the Regional Ethics Review Board in Stockholm (Dnr 2011/570-31/5, 2011/1425-31, and 2012/1107/32). We approached all 32,005 twins in the cohort with a letter sent to their residential addresses, which contained a brief description of the study, a statement that their participation was voluntary and may be discontinued at any time, and that their commencing the web survey would constitute giving informed consent. The letter contained an individual pass code that the participants used to log in to the web survey. The full sample that completed the present wave consisted of 11,543 twins, 5,651 singletons and 5,892 that were part of a complete twin pair, comprised of 6,651 females and 4,892 males, with ages between 27 and 54 at the time of responding to the survey (*M* = 40.7, *SD* = 7.75).

### Variables

The survey contained a wide range of instruments and questions that took between 55 and 90 min to complete, the procedures of which are further described in Appendix 1.3. Many of these instruments are described in previous publications (e.g., Mosing et al., 2014a,b, 2015; Ullén et al., 2014, 2015; Theorell et al., 2015). Of these, only auditory SRT, sex, and birth year were utilized in the present study.

The auditory SRT task was implemented both as a Flash and as a Shockwave application, as detailed in Appendix 1.1. Auditory stimulation was chosen because the control and latency of sound presentation varies less across different computer systems than visual presentation. Shockwave had superior temporal accuracy and was used if it was installed on the participant’s computer. Otherwise, the participant was given the option of installing Shockwave. If the participant declined or the Shockwave installation failed, the Flash application was used. If Flash was not installed and the participant did not agree to install it, no SRT data could be obtained and that participant was excluded from the analysis. The SRT datum for each participant was the median of 25 trials, corrected for known differences between Flash and Shockwave, different browsers, operating systems, and so forth, in order to increase inter-individual reliability as described in Appendix 1.2.

Conscript intelligence test scores were obtained from mandatory military testing that the males in the present study underwent when they were about 19 years old. The purpose of obtaining these was to assess possible effects of cognition on attrition, specifically if there was any cognitive ability × birth year interaction that might be an alternative explanation for secular changes in the SRT measure. The nature of these military intelligence tests are secret, so as to avoid their dissemination and hence cheating or other validity threats. The authority releases no information about the items, but does release the maximum score and the raw scores for each individual. Test results were only available for the subset of the male cohort born between 1959 and 1976, because systematic military psychometric testing was gradually discontinued in the late 1990s.

## Results

Of the total 32,005 twins that were invited to participate, 11,543 logged in and responded to at least one item after receiving up to three surface mail reminders, and 7,081 completed the SRT tests. First, intra-individual SRT reliability was investigated by correlating the medians for the first 12 and the second 13 SRTs for each participant. As this value was as high as 0.953 we did not attempt to correct for it in the following analyses. **Table 1** shows the descriptive statistics of the SRT data.

**Table 1:** Descriptive data for SRT, 1959–1985.

	SRT		
Sex	*N*	*M*	*SD*
Females	4,084	243.9	47.5
Males	2,997	239.5	48.4
Both	7,081	241.0	47.1

Second, we considered SRTs as a function of birth year, and found a rather steep increase in the general level of about 5 ms for the four oldest birth years (1959–1962) when compared to the next oldest (1963–1968). This is consistent with significantly slowing auditory SRT in cross-section only from about 50 years of age (Der and Deary, 2006; Jaworski et al., 2011). Participants that represent earlier-born genotypes – expected to have faster reaction times – will also exhibit slower performance due to phenotypic deterioration. Aging effects can in principle be controlled for on the basis of longitudinal studies, which follow the same genotypes as they age. A reasonable estimate of the age-related decline for auditory SRT is an approximately linear increase of 0.5 ms per year of age from young adulthood to late middle age (Koga and Morant, 1923; Fozard et al., 1994). Those samples were relatively small, about 50–150 individuals per decade, and may therefore not be able to detect non-linearity in the trend. Der and Deary (2006) found, with about 1,000 individuals per decade, both quadratic and cubic trends in visual SRT as a function of age, indicating accelerating decline above about 50 years of age. Any substantial effect of these trends on the SRT magnitude does not seem to appear before 55 years, however (Der and Deary, 2006, pp. 65–66). Thus, the documented aging effects are somewhat ambiguous. The oldest participants in the present sample were 53 years old, and are therefore so close to the suggested breakpoints that decisions to adjust or to exclude cohorts are difficult. We therefore present the data both with and without a linear adjustment of 0.5 ms per year, and perform regressions both for the full data set and with the four oldest age cohorts (+50 years) excluded. This amounts to four distinct trend models.

**FIGURE 1:**
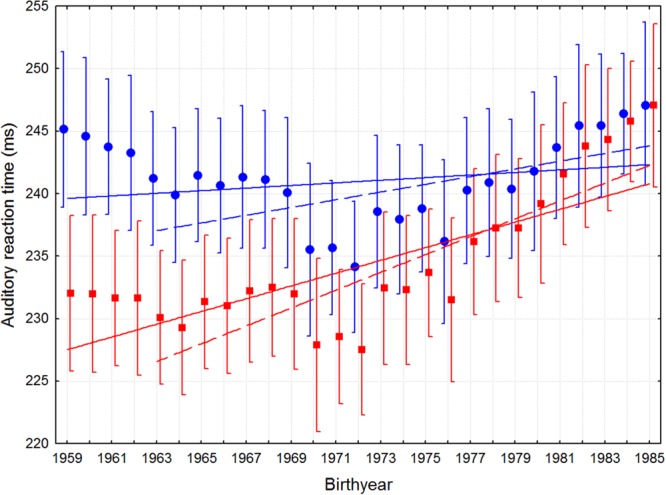
**Mean auditory SRT as a function of birth year with 95 percent confidence intervals.** The filled circles depict the mean non-adjusted SRTs, and the squares the aging-adjusted SRTs. Linear regression lines are fitted to the data, whose parameters are given in **Table 2**.

**Figure 1** shows the secular change in SRT across the 27 years, with fitted linear trends for both birth years 1959–1985 and 1963–1985, and for both aging-adjusted and non-adjusted data. There are indications of an accelerating slowing from about 1970. Regression analyses, detailed in **Table 2**, show that, for both sexes together, all temporal trends starting between 1963 and 1985 are significantly different from zero, whereas only the non-adjusted 1959–1985 trend is not.

**Table 2:** Regressions of auditory SRT on birth year for 1959–1985 and 1963–1985, for aging-adjusted and non-adjusted SRTs, and for females, males, and both sexes together.

Year range	*N*	Intercept	Slope	*SE*	*T*	*R*^2^	*p*
**Non-adjusted data, both sexes**							
1959–1985	7,081	163	0.040	0.073	0.54	<0.001	0.580
1963–1985	5,966	-220	0.233	0.091	2.57	0.001	<0.01
**Aging-adjusted data, both sexes**							
1959–1985	7,081	-828	0.539	0.073	7.41	0.008	<0.00001
1963–1985	5,966	-1212	0.730	0.091	8.07	0.011	<0.00001
**Non-adjusted data, females**							
1959–1985	4,084	284	0.021	0.095	0.22	<0.001	0.826
1963–1985	3,427	-10.6	0.128	0.118	1.08	<0.001	0.278
**Aging-adjusted data, females**							
1959–1985	4,084	-708	0.479	0.095	5.05	0.006	<0.00001
1963–1985	3,427	-1003	0.630	0.118	5.31	0.008	<0.00001
**Non-adjusted data, males**							
1959–1985	2,997	-11.2	0.127	0.113	1.12	<0.001	0.262
1963–1985	2,539	-503	0.375	0.141	2.66	0.003	<0.01
**Aging-adjusted data, males**							
1959–1985	2,997	-1002	0.630	0.113	5.53	0.010	<0.00001
1963–1985	2,539	-1494	0.870	0.141	6.19	0.015	<0.00001

When separated by sex, the non-adjusted trend for males was significant when the 50+ participants were excluded, but not for the females. To examine the robustness of the females’ non-adjusted trend, regressions were computed for each span from 1959–1985 to 1973–1985, revealing that all trends starting from 1968 or later were significant for the females. With regards to the influence of computer system-related bias, the corresponding increasing trends were somewhat larger for the uncorrected SRT data (see Appendix 1.2).

It could be argued that participants approach the task differently as a function of age, and that this might constitute a measurement error that confounds a real secular slowing in SRT. For example, younger participants might tend to have poorer impulse-control or be more competitive, which would lead them to ‘jump the gun.’ In this case, such behavior should result in larger performance variability within these cohorts. We therefore computed the SD of SRT across participants in each birth year cohort, which, contrary to predictions from this hypothesis increased with age, from 37 ms for those born 1985 to 53 ms for those born 1959 (Appendix Table A1). To summarize this result, the correlation between birth year and SRT SD was -0.318 (*N* = 27, n.s.), consistent with typical effects of aging (Deary and Der, 2005).

It could also be argued that differential attrition as a function of birth year might cause the increase in SRT. This would be the case if there were a tendency for slower SRT individuals to dropout to a greater degree among the older cohorts, perhaps due to factors such as mortality (e.g., Deary and Der, 2005). This possibility was examined by comparing cross-sectional trends among both the participants’ and the dropouts’ general intelligence test scores. The validity of this approach is based on the fact that less intelligent individuals are more likely to drop out from studies in general (e.g., Deary and Der, 2005, p. 193), and SRT and *g* are related to one another genetically (Madison et al., in press) and through a common phenotypic chronometric *g*-factor (Verhaeghen and Salthouse, 1997). Scores on the Swedish Enlistment Battery (SEB) during conscription at 19 years of age were used for this purpose. These data were available only for the males who took the test in the interval 1977–1997, corresponding to birth years 1959–1979. The mean number of individuals per birth year was 127.3 for the participants and 201.0 for the dropouts, with a total of 2,292 participants and 3,619 dropouts. However, the test versions administered in this interval – SEB-67 (1970–1979), SEB-80 (1980–1993), and CAT-SEB (from 1994) – differ in their structure (Carlstedt, 2000), which incurs a risk of incorrect imputation of secular trends. We therefore performed linear *N*-weighted regressions only for the SEB-80, because it covered the largest numbers of years in the period for which data were available. Significant annual score point increases in intelligence were found among the participants (β = 0.085, *N* = 1,739, *p* < 0.00001) as well as among the dropouts (β = 0.088, *N* = 2,847, *p* < 0.00001), as expected based on the Flynn effect (Flynn, 2013). The difference between these trends was however not significant, as the lower confidence interval of the slope for the participants, which was the largest (0.0036), overlapped with the slope for the dropouts. Thus there was no significant tendency for older dropouts to be less intelligent than younger ones, suggesting that attrition is not likely to be generating the observed trends, at least among the male participants and inasmuch as general intelligence serves as a useful proxy for processing speed (e.g., Verhaeghen and Salthouse, 1997; Jensen, 2006).

## Discussion

We found clear trends toward slowing auditory SRT when birth year was regressed against year-on-year SRT means for the years 1959–1985. It is notable that even without adjustment for aging, the SRT speed of the oldest participants is about the same as that of the subsequent generation, whom in the late twenties are supposed to have the shortest SRTs of all age groups (Der and Deary, 2006). Before discussing the implications of these results further, we consider possible confounds and sources of error.

First, online data collection provides much less control than in laboratory measurements. Specifically, we could not assess participants’ hearing or fulfillment of the task instructions, although in the case of age-related changes hearing, this would clearly have a *suppressing* effect on the secular trends observed, in as much as older people have less acute hearing than younger people. Differences in participant computer systems could only partly and indirectly be controlled, by recording their system specifications and comparing results across system components, such as types and versions of operating systems, web browsers, and audio–visual media plug-ins. It is clear that keyboards, sound cards, mice and other hardware components also vary in terms of the lags and performance variances that they add to the speed measurements (Woods et al., 2015). Some *post hoc* corrections were applied to the SRT data, as described in Appendix 1.2. Whilst they decreased the inter-participant variability, the slowing trend was nonetheless robust across SRT data, both corrected and uncorrected for computer system-related variability.

The validity of the SRT data as a measure of general intelligence stems from the finding of a -0.18 correlation with IQ in this sample, despite possible unsystematic error (Mosing et al., 2015). That this correlation is smaller than those values typically found in laboratory studies is consistent with the larger error associated with on-line measurement of both SRT and IQ. The few studies that have correlated auditory SRT task performance with pencil-and-paper measures of IQ have reported correlations between -0.40 to -0.30, using Raven’s SPM (Poon et al., 1986; Agrawal and Kumar, 1993), and -0.05 when the Raven’s SPM Plus was employed (Holm et al., 2011). Note that the validity threat in the present study is bias rather than random error, and that a range of possible sources of bias that might be related to birth year were controlled.

Second, longitudinal attrition is a potential source of bias, in as much as there exists a positive correlation between IQ and longevity and a positive correlation between IQ and willingness to participate in research. We were however able to assess such effects using conscript IQ data obtained 18–36 years earlier. Older male dropouts did not exhibit lower means of general intelligence than younger ones, neither were their cross-sectional characteristics significantly different from those of the participant group. Thus, any true difference between participants and dropouts in terms of a potential age-related decline in *g* (and by proxy processing speed) is unlikely to have generated the secular increase in SRT. That there was nevertheless a difference which went in the opposite direction makes our estimate of secular SRT increase all the more remarkable. As an additional control, we compared the variability of the dropouts and the participants on the *g* measure as a means of estimating indirect range restriction, and found that the SD difference was extremely small (0.084) as well as non-significant, despite the large numbers of participants. The difference is nonetheless in the expected direction, that is, larger for the dropouts, but since it is about 1.5% of the mean SD it must be deemed inconsequential. No correction for restriction of range was therefore performed. Both these calculations were based on males, but it seems reasonable to assume that similar trends would apply for the females, had data been available for them. These calculations rely on the use of the *g* scores of the attrition sample as a proxy for their SRT scores. If attrition characteristics for different cognitive ability measures are substantially discrepant then this assumption would not hold.

Third, we cannot account for the shorter SRTs for participants born 1970–1971. It could be a continuation of the 1972–1985 trend, followed by aging related increase from 1969–1959, but could also represent some random variability. The regressions are however fitted to all years, except for the 1963 cutoff, and are therefore conservative with respect to the possibly much more dramatic trend suggested in 1972–1985.

Thirdly, we assume that the present sample is representative of the general population. The Swedish Twin Registry (STR) contains all twins born in Sweden, so the remaining issues are whether twins and non-twins or participating and non-participating twins differ in some aspect that might interact with age cohort and SRT speed. On the first count, we are not aware of any way in which twins’ reaction times would change with birth year in any other way than would be the case for people in general. Indeed, STR data are commonly generalized to the general population across a wide range of variables including, for example, body height and educational attainment (Magnusson et al., 2006), criminality and psychiatric disorders (Webb et al., 2011), and political attitudes (Dawes et al., 2014). On the second count, it has been argued that more genetically similar individuals are also treated similarly, thus violating the equal environments assumption. A large cross-national study using misclassified twins showed the equal environments assumption seems to hold however (Conley et al., 2013).

In the present study, the secular slowing trend was present in all cohort comparisons (males, females, and both sexes combined), and was significant across the entire range of birth years for both the males and the whole sample, but not for the females, who nonetheless exhibited an overall negative trend in SRT performance consistent with potential secular slowing. A test of robustness involved comparing birth years from 1963–1985 for the males and 1973–1985 for the females, and the whole sample, in order to determine the stability of the secular trend. For the whole sample and for the males significant differences were found for all comparisons. For females, significant slowing was observed for birth year ranges from 1968–1985 to 1973–1985, suggesting some trend-robustness also within this subgroup.

A potential cause of the apparent slowing may be exposure to neurotoxic industrial by-products such as heavy metals (Silverman, 2010) and dioxins (ten Tusscher et al., 2014), which may reduce SRT performance via their effects on peripheral nerve conduction velocity. However, as Silverman notes, known neurotoxins have come under tight governmental regulation, emissions have tended to decrease, and serum levels of lead, for example, have decreased since 1970 in the USA (Silverman, 2010, p. 46).

Another possible cause of this trend may be relatively recent micro-evolutionary trends favoring lower *g* in the population of Sweden. Several studies have revealed that *g* and fertility are inversely related in the US and the UK (as reviewed in Woodley of Menie, 2015) among cohorts born as far back as the 1890s (Lynn and Van Court, 2004; Lynn, 2011). However, the relationship between *g* and fertility in Scandinavian countries is less well characterized. Only one study has attempted to examine these trends across birth cohorts in Sweden (Vining et al., 1988). Utilizing aggregate data on fertility and IQ for a mixed-sex sample of Swedish cohorts resident in Stockholm county and born between 1909 and 1940 from Vining et al. (1988), it was possible to reconstruct predicted generational changes in genotypic IQ (i.e., the heritable variance component of IQ) due to the changing patterns of selection (i.e., the correlation between IQ and fertility established for each cohort) for four cohorts (see Appendix 2 for details of the method).

**FIGURE 2:**
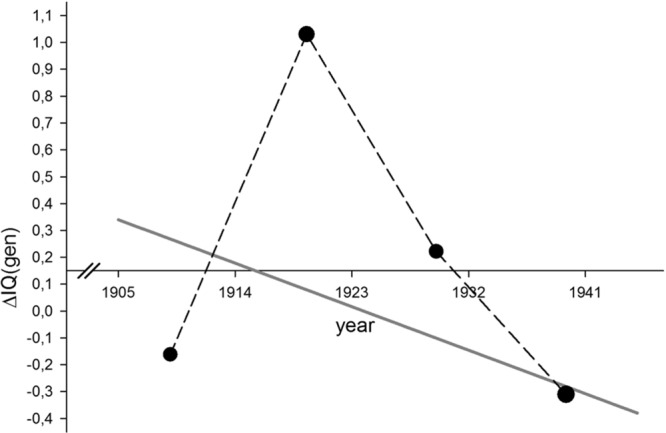
**Predicted generational change in Swedish genotypic IQ estimated in four mixed-sex birth cohorts (1909, 1919.5, 1929.5, and 1936.5).** The overall *N-*weighted temporal trend across these four values is also shown (gray line).

**Figure 2** reveals considerable inter-cohort variability in the degree to which genotypic IQ would be expected to change per generation, however there is a clear secular trend toward the IQ change becoming increasingly more negative with time (*r* = -0.377, *p* < 0.05, *N* = 1,746). This suggests that Sweden transitioned into a selection regime favoring lower levels of genotypic IQ somewhere between 1915 and 1930. The oldest cohorts (those born in the late 1950s) would have had parents born in the late 20s and early 30s (assuming a generation length of 2.8–3 decades), thus they would have been born on the cusp of the transition. Subsequent cohorts would have had parents born at a time when microevolutionary trends should have been unambiguously favoring lower genotypic IQ, based on the temporal trend.

Consistent with these observations, Emanuelsson et al. (1993) found evidence that the Flynn effect had leveled off in Swedish cohorts born between 1972 and 1977. A recent study of Swedish 18- to 19-year-old military conscripts evaluated between 1992 and 1993 (born 1973–1974) demonstrates the first indication of an anti-Flynn effect in Sweden, amounting to loss of -0.26 IQ points per decade (Rönnlund et al., 2013). Our finding of slowing SRT could therefore be considered consistent with these findings of stalling Flynn effects and even secular losses among younger Swedish cohorts.

## Conclusion

An increasing number of studies converge in indicating secular IQ declines in several geographical regions, using different strands of evidence. The present study has, for the first time, documented a potential secular slowing of SRT in Sweden. More importantly, it employed a measurement design that avoids some of the weaknesses in previous studies as discussed in Dodonova and Dodonov (2013). That we essentially replicated previous results provides multi-method validation, which should motivate further study into the reasons for, and possible consequences of, these trends.

## Author Contributions

GM and MW conceived the study and wrote the paper, and GM and JS analyzed the data.

## Conflict of Interest Statement

The authors declare that the research was conducted in the absence of any commercial or financial relationships that could be construed as a potential conflict of interest.
