# Supplementary material for: Secular Slowing of Auditory Simple Reaction Time in Sweden (1959–1985)
Source: Front Hum Neurosci. 2016 Aug 18;10:407. doi: 10.3389/fnhum.2016.00407 (PMC4988978; doi:10.3389/fnhum.2016.00407)
Supplement: Supplementary file 1 [file Data_Sheet_1.DOCX]

Appendix 1. Details of methods for collecting and correcting SRT data

A1.1 SRT measurement

As mentioned in the Method section, the SRT task was implemented both as a Flash and as a Shockwave application, the user interfaces of which were identical. It appeared as a white box in the centre of the browser window, approximately 100 mm tall and 150 mm wide, depending on the size of the screen. General instructions appeared in black font in the left part of the application window (here translated from Swedish): “*Your task is to press the space bar as fast as possible after each sound. Use your best/dominant hand. Wait until you hear the sound, and then press the space bar. Click ‘Start’ to begin the test*”. When the participant started the first trial there was a silent interval randomly varied from a rectangular distribution from 1.5 to 3.5 seconds before the stimulus was presented through the computer’s speaker or headphones, whichever was connected. The stimulus consisted of a cow-bell sound, whose loudness depended on the equipment and the participant’s settings in the previous set-up stage. An approximately 20 mm tall and 60 mm wide green box with the text “Press the spacebar!” appeared simultaneously with the sound in the middle of the application window, and disappeared when the bar was pressed, which issued the next random interval, and so forth. The application recorded the time interval from the stimulus presentation to the space bar response and stored it in temporary memory until the application ended, when RTs from all trials were sent to the server. The application was run twice, first with 10 trials for training and then with 25 trials for the task proper, the durations of which were about 30 s for the first and 75 s for the second task. The general instructions were repeated between runs.

A1.2 SRT data correction

The SRT datum for each participant consisted of the median of the 25 RTs from the second task, which eliminates the influence of occasional long delays that typically appear in multi-tasking computer systems (Wallace & Madison, 2012).

Because of the variety of participants’ computer systems and configurations, an extensive analysis of possible systematic effects was performed (Madison, Cassel, & Ullén, in preparation). That analysis was based on the so-called user strings obtained from the system through the browser, which provides code that designate, among other things, the operating system (OS), browser, and multimedia plug-in type and version numbers. If there were some systematic relationship between computer system properties that affect the median RT interval as a function of age or sex, then this confound could bias the present results. For the sake of transparency, we will here provide an overview of the influence of equipment, and the general measures taken to correct for it. Before this, however, we should point out that we ran the analyses of the whole sample on both corrected and uncorrected SRT data, the latter of which produced somewhat greater slopes, which means that the reported secular RT increase is not an artefact of the data manipulation. The major effects of the correction were rather to decrease inflated RTs due to system delay by about 30 ms on average, and decrease the mean inter-individual SD within each age cohort from 90.6 to 47.9 ms.

First, as regards numbers, 1,111 of Windows users had Explorer, 610 had Firefox, 559 had Chrome, 13 had Opera, and 9 had Safari installed as their browser. Among Mac OS users, 471 had Safari, 152 had Firefox, and 72 had Chrome. The OS versions for PC computers running Shockwave were Windows 7 (2,326), XP (696), Vista (639), and Windows 8 (53). The OS versions for PC computers running Flash were Windows 7 (1,578), XP (383) Vista (312), and Windows 8 (54). Apple computers had Mac OS versions 10.5 to 10.8.2, 234 with Shockwave and 697 with Flash. No significant mean RT differences were found between Mac OS versions running either Flash or Shockwave, partly owing to small N in the range 9 – 171. There were only 44 participants using the Linux OS.

The analyses uncovered several substantial effects of system components. Windows versions with Flash yielded 50 – 85 ms longer mean RTs than both Windows with Shockwave and Mac OS with either Flash or Shockwave. Chrome produced 180 - 195 ms longer mean RTs in both Windows and Mac OS than did all other browsers, which affected 631 cases out of the total 7,081 that completed the RT test. In addition, smaller but statistically significant differences were found between Windows versions. Compared to Windows XP, the Shockwave application produced longer mean RTs in Windows 7 (21 ms), Windows 8 (24 ms), and Vista (27 ms). Compared to Windows 7, the Flash application produced longer mean RTs in Vista (13 ms) and XP (17 ms). No other mean differences for either Windows or Mac OS were significant.

To control for the computer system-related variability, statistically significant differences for each multimedia plug-in and OS combination were subtracted from the raw RT data, in addition to a general system delay obtained by a benchmark test run on eight Windows computers with the Shockwave application, as described in a forthcoming paper (Madison, Cassel, & Ullén, in preparation).

In conclusion, the applied corrections primarily calibrate the SRT estimates closer to their true magnitudes and thereby decrease mean inter-individual within-year cohort variability by 47%. Although this is useful for the individual and small group level, it had no substantial influence on the secular trends presented here, as evidenced by comparing corrected and uncorrected data.

| Table A1. Descriptive SRT statistics for each birth year cohort, corrected for computer system-related variability but not adjusted for ageing. | | | | | | |
| --- | --- | --- | --- | --- | --- | --- |
| Birth year | *N* |  | *SD* |  |  |  |
| 1959 | 284 | 245.123 | 53.746 |  |  |  |
| 1960 | 294 | 244.598 | 54.767 |  |  |  |
| 1961 | 269 | 243.744 | 46.619 |  |  |  |
| 1962 | 250 | 243.250 | 50.406 |  |  |  |
| 1963 | 309 | 241.205 | 48.765 |  |  |  |
| 1964 | 287 | 239.889 | 46.398 |  |  |  |
| 1965 | 328 | 241.456 | 49.034 |  |  |  |
| 1966 | 280 | 240.632 | 45.902 |  |  |  |
| 1967 | 266 | 241.325 | 47.175 |  |  |  |
| 1968 | 263 | 241.124 | 45.390 |  |  |  |
| 1969 | 229 | 240.062 | 46.245 |  |  |  |
| 1970 | 226 | 235.503 | 52.812 |  |  |  |
| 1971 | 270 | 235.662 | 44.904 |  |  |  |
| 1972 | 271 | 234.141 | 43.791 |  |  |  |
| 1973 | 231 | 238.546 | 47.106 |  |  |  |
| 1974 | 266 | 237.920 | 49.471 |  |  |  |
| 1975 | 244 | 238.801 | 40.402 |  |  |  |
| 1976 | 228 | 236.154 | 50.292 |  |  |  |
| 1977 | 216 | 240.245 | 43.582 |  |  |  |
| 1978 | 234 | 240.871 | 45.835 |  |  |  |
| 1979 | 298 | 240.369 | 48.813 |  |  |  |
| 1980 | 248 | 241.795 | 50.651 |  |  |  |
| 1981 | 286 | 243.685 | 48.761 |  |  |  |
| 1982 | 256 | 245.424 | 52.837 |  |  |  |
| 1983 | 273 | 245.429 | 47.955 |  |  |  |
| 1984 | 318 | 246.378 | 43.621 |  |  |  |
| 1985 | 129 | 247.186 | 37.539 |  |  |  |
| Sum, M | 7,081 | 240.702 | 47.176 |  |  |  |
|  | | | | | | |

A1.3 *Procedure*

All instructions were administered in Swedish, and are conveyed in translation below. The survey began with 17-37 items about music experience and possible music training, depending on branching due to responses to earlier items, and another 117-123 items including sports activities, personality, and motivation. After this came a 24 item matrix reasoning intelligence test, which was implemented in Flash, followed by 41 items about occupational preferences and further personality traits, 6 items about demographic variables, 20 items about emotional reactions, and 12 items about health. The SRT test came next, and was introduced thus: “*Now follow a number of tests that measure your reaction time and time precision by pressing keys in relation to sounds that you will hear. The computer you use must therefore be able to play sounds through loudspeakers or headphones, and that is the first thing that will be tested. It is important that you closely follow the instructions when you later perform the different tests. Please therefore attend carefully to the instructions and the description of what the different signals mean before each test. These tests take about 15 minutes, so please make sure you have this time at your disposal without distractions before you start. Press the keys normally, as when you type, that is, press them down briefly and do not keep them depressed*”. After this a Flash application tested the sound reproduction and the key functions of the computer, and branched further to various routes for installing the Shockwave or Flash software, if required. The SRT measurement procedure specifically was described under A1.1 above.

Appendix 2. Details of methods for estimating genotypic IQ change in Sweden

Inverted *N*-weighted analysis will be utilized to reconstruct the correlations between IQ and fertility or sibling numbers in these datasets, and also to obtain the IQ change expected on the basis of selection, using aggregated data from Vining et al. (1988). Inverted *N*-weighted analysis simply involves fixing the values of the independent and dependent variables with the class or sub-class aggregate values for both, and then replicating the value pairs by the sample size in order to create the pools for each class and sub-class. In the case of Vining and co-workers (1988), the dependent class variable is birth years, the sub-class variable is aggregate IQ and the independent variable is aggregate fertility, thus for the year 1920 (mid-range of 1915-24), we assign the (male) fertility value of 1.65 and an IQ value of 100 (mid-range of 95 and 105) to 50 cases (the sample size). Repeating this for each IQ grouping gives us the sub-class pools for the entire 1920 male class. In Excel the sub-class pools are delimited with cells. Simply correlating IQ and fertility across the sub-class pools gives us the equivalent of the *N*-weighted correlation between fertility and IQ for each class (i.e. birth year), which approximates the result of the primary analysis involving the raw data (i.e. as if we had every individual IQ and fertility datum in the analysis).

In the case of the Swedish data, the lowest and highest IQ sub-classes are listed as <94 and >125 respectively. To assign a mid-range value to these sub-classes, we take the difference between IQ values across the subclasses that report an upper and lower range (14), subtract this value from the lower and add this value to the upper sub-class values (i.e. 94-14 and 125+14 respectively), thus simulating lower and upper range values for these sub-classes (80 and 139 respectively). The mid-range value can then be used as the sub-class value in these cases (87 and 132 respectively).

Inverted *N*-weighted analysis also allows us to determine the generational change in IQ due to selection. In order to estimate this, the selection strength (*S*) needs to be determined. This is achieved by taking the difference between the average IQ of the sample and the weighted average IQ of the sample, weighting by either numbers of siblings or offspring. This difference constitutes the selection pressure strength operating on IQ over a generation, scaled in terms of the expected change in *phenotypic IQ* (Lynn, 2011). The pooled sub-classes can be used to reconstruct both parameters necessary for computing *S*, by averaging across the pooled sub-class IQs, and then taking the weighted average IQ across the pooled sub-classes, weighted by the fertility values. Again, these values should be similar to those that would be obtained from primary analysis of the raw data.

Based on the *Breeders Equation*, multiplication of *S* by the additive heritability of general intelligence (the value of .86 is chosen based on a latent variable model conducted by Pannizon et al., 2014) yields the degree to which IQ would be expected to change in response to selection, or *R* (Lynn, 2011).

As the Swedish data were collected over four different birth year classes, *N*-weighted temporal correlation analysis can be employed in order to determine whether there are temporal trends in the pattern of selection operating on genotypic IQ. This correlation can be computed by assigning to each individual in a birth-year class that class’ male and female combined value of *R*, and then correlating these pooled *R* values with birth year. As there appears to be no consistent secular trend in the heritability of IQ in Scandinavia (Sundet, Tambs, Magnus & Berg, 2002), the same *h^2^* value (i.e. 0.86) was assigned to all birth years. All values are reported in Table A2.

Table A2**.** Sample sizes, reconstructed correlation between fertility and intelligence for the male, female and combined samples, selection gradient (phenotypic IQ change; *S*) and genotypic IQ change (*R*). *N* = 854 females and 892 males.

| **Birth year** | **Male *N*** | **Male *r*_(IQ_** × **_Fert.)_** | **Female *N*** | **Female *r*_(IQ_** × **_Fert.)_** | **Combined *r*_(IQ_** × **_Fert.)_** | ***S*** | ***R*** |
| --- | --- | --- | --- | --- | --- | --- | --- |
| 1909 | 177 | 0.459 | 179 | -0.869 | -0.209 | -0.188 | -0.162 |
| 1919.5 | 219 | 0.959 | 246 | 0.241 | 0.579 | 1.199 | 1.031 |
| 1929.5 | 180 | 0.406 | 200 | 0.353 | 0.378 | 0.258 | 0.222 |
| 1939.5 | 278 | -0.837 | 267 | 0.233 | -0.313 | -0.361 | -0.31 |
| Total (*N*) | 854 |  | 892 |  |  |  |  |
